# Prevalence of Self-Reported Swallowing Difficulties and Swallowing-Related Quality of Life Among Community-Dwelling Older Adults in India

**DOI:** 10.1007/s00455-024-10696-0

**Published:** 2024-04-18

**Authors:** Thejaswi Dodderi, Drishti Sreenath, Mahima Jayaram Shetty, Uzair Chilwan, Santosh P. V. Rai, Sheetal Raj Moolambally, Radish Kumar Balasubramanium, Mohit Kothari

**Affiliations:** 1 Department of Audiology and Speech Language Pathology, Kasturba Medical College, Mangalore, Manipal Academy of Higher Education, Manipal, India; 2 Department of Radiodiagnosis and Imaging, Kasturba Medical College, Mangalore, Manipal Academy of Higher Education, Manipal, India; 3 Department of General Medicine, Kasturba Medical College, Mangalore, Manipal Academy of Higher Education, Manipal, India; 4 Hammel Neurorehabilitation Center and University Research Clinic, Department of Clinical Medicine, Aarhus University, Hammel, Denmark

**Keywords:** Dysphagia, Swallowing, Older adults, EAT-10, DHI

## Abstract

Self-reported swallowing difficulties are highly prevalent but underreported among older adults. The aging population in India is increasing, yet there is a lack of empirical data on self-reported swallowing difficulties in older adults. In the present study, we aimed to estimate the prevalence of self-reported swallowing difficulties and assess the swallowing-related quality of life (QOL) among community-dwelling older adults in India. We recruited 361 older adults (60–91 years) from the community. Participants completed the Eating Assessment Tool-10 (EAT-10) to assess self-reported swallowing difficulties and the Dysphagia Handicap Index (DHI) to assess swallowing-related QOL. Participants rated the EAT-10 from 0 for ‘*no problem*’ to 4 for ‘*severe problem*’. The DHI rating included 0 for ‘*never*’, 2 for ‘*sometimes*’, and 4 for ‘*always*’. The total scores of EAT-10 and DHI were summarised using descriptive statistics. Statistically significant differences between pass–fail groups of EAT-10 and DHI were evaluated using an independent *t*-test and multivariate analysis of variance test, respectively. The overall mean score for EAT-10 was 3.34, and 7.56 for DHI, with higher scores observed among females. 36.6% of older adults self-reported experiencing swallowing difficulties, while 47.4% self-reported having poor swallowing-related QOL at *p* < 0.05. A strong positive correlation (*r* = 0.86) was found between EAT-10 and total DHI scores at *p* < 0.001. The present study sheds light on the widespread yet underreported issue of self-reported swallowing difficulties and the impact on swallowing-related QOL among older adults in India. These findings emphasize the urgent need for early swallowing screening programs among older adults.

**Supplementary Information:**

The online version contains supplementary material available at 10.1007/s00455-024-10696-0.

## Introduction

Swallowing difficulties in older adults (individuals aged >60 years) has been recognized as an ‘*geriatric syndrome*’ affecting 40 million individuals in Europe [1]. Aging is an independent predictor of swallowing difficulties and has 20% higher risk in the population above of 65 years old [2]. Swallowing difficulties and aging may lead to health issues like aspiration pneumonia, reduced nutrition, and dehydration [3–5], resulting in extended hospital stays and economic burden [6]. Thus, early identification and intervention for swallowing difficulties in older adults are crucial for promoting active aging. Video-fluoroscopy study of swallowing quantifies physiological variations observed in oral and pharyngeal regions among older adults [7]. It provides valuable insights for Speech-Language Pathologists in clinical decision-making. However, it does not provide insight into patients’ day-to-day challenges related to swallowing difficulties. Further, video-fluoroscopy studies are confined to objective data and do not assess the broader impact of swallowing difficulties on individuals’ health and their quality of life (QOL). In response to these limitations, self-reported measures have become an essential tool in the dysphagia practice.

The primary focus of self-reported measures are to gather reliable information directly from patients to help in shared treatment decisions [8], and to understand the psychosocial burden associated with health ailments [9]. Speech-Language Pathologists have used different self-reporting questionnaires such as the Eating Assessment Tool (EAT-10) [10–17], Dysphagia Handicap Index (DHI) [18], Sydney Swallow Questionnaire [19], a combination of EAT-10 and Sydney Swallow Questionnaire [20] and M.D. Anderson Dysphagia Inventory [21] to identify individuals at risk for swallowing difficulties [22]. These questionnaires are popular for brevity and highly valued among clinicians due to their cultural adaption in different languages [23, 24]. The risk of swallowing difficulties in older adults have also been extensively studied using these self-reported measures [25], adding to our understanding of this neglected health issue [26].

The global estimate of swallowing difficulties among adults is reported as 43.8%, and this estimate increases to 48.1% in older adults [27]. Studies conducted over the years suggest that self-reported swallowing difficulties among community-dwelling older adults ranges from 9.8% to 53.8% [10, 12, 13, 15, 16, 28–31]. According to a systematic review and meta-analysis of the literature, the prevalence of swallowing difficulties in community-dwelling older adults falls within the range of 5–72% [32]. Prevalence rates of self-reported swallowing difficulties in older adults vary depending upon: (1) settings—these rates range from 31.9% [33] to 77% [34] in hospitals, and from 14.9% [35] to 68% [36] in nursing facilities; (2) questionnaire used—23.7% of individuals reported difficulties when using the EAT-10 questionnaire, while only 9.49% reported issues on the Sydney Swallow Questionnaire [20], and (3) country-specific—the prevalence rates are 2.6% in United States [25], 10.6% in Canada [2], 22.1% in New Zealand [14], 25.1% in Japan [13].

Despite the wealth of studies across different domains and regions, there is currently no available data on self-reported swallowing difficulties for older adults within Indian population. Limited information suggests that 2% of older adults in India require full or partial assistance for feeding [37]. To the best of our knowledge, there is no shortage on availability or accessibility of self-reporting swallowing questionnaires in regional languages of India for research purposes or measuring clinical outcomes [38–42]. However, for reasons not known to us, no previous attempts have been made to estimate the prevalence of self-reported swallowing difficulties among community-dwelling older adults in India. Considering the estimated 41% increase in the number of older adults in India in 2021, reaching 138 million [43], there is an urgent need to assess prevalence of self-reported swallowing difficulties among community-dwelling older adults aged >60 years in India. The findings of this study contribute valuable information for healthcare practitioners, aiding them in better understanding the scope of this issue within community-dwelling older adults in India. This can also prompt the integration of systematic screening protocols, such as the EAT-10 and/or DHI, into routine assessments to identify older adults with swallowing difficulties early.

The purpose of our study was to estimate the prevalence of self-reported swallowing difficulties and assess swallowing-related QOL in community-dwelling older adults of India. Additionally, we aimed to investigate whether swallowing-related QOL differed among older adults with and without self-reported swallowing difficulties.

## Method and Materials

### Study Design

The present study was undertaken in Mangalore (Karnataka, India). The study followed a cross-sectional design and non-probability purposive sampling. The research proposal received ethical clearance from the Institute Ethics Committee (approval number IEC KMC MLR 05-2021/171). The data of present study is derived from Phase I data of another overarching study on community-dwelling older adults by the research team. The survey utilized a descriptive paper design to estimate period prevalence data, collecting data between February 2022 and March 2023.

### Participants

The study consisted of 361 older adults (178 females) aged between 60 and 91 years with mean age of 71.09 ± 8.45 years who were purposively sampled from the community. The sample size was determined based on a prior nationwide survey in New Zealand that utilized convenience sampling to estimate self-reported swallowing difficulties in community-dwelling older adults (*n* = 1020) aged 65–96 years [14]. The sample size formula used was *n* = *Z*^2^*pq*/*d*^2^, where *Z* = 1.96 at 5% level of significance, *d* = 5% margin of error, *p* = prevalence of 22.1% [14]. Accordingly, a sample size of 271 older adults was necessary to estimate prevalence of self-reported swallowing difficulties and swallowing-related QOL among older adults in India. The study included older adults who lived independently within city limits of Mangalore (Karnataka, India). Further, only older adults who could read either English or Kannada were included in the study. Individuals with self-reported history of head, neck, and other cancer, neurological disease, swallowing impairment, congenital visual impairment, or those presently dependent on non-oral feed were excluded. Participants who self-reported a history of surgery to the oral cavity and pharyngeal and laryngeal structures were also excluded. Prior to data collection, participants provided written informed consent.

### Measurement Tool

The EAT-10 and DHI questionnaire were used to assess self-reported swallowing difficulties and swallowing-related QOL, respectively. The selection of these questionnaires was motivated by their availability in various regional languages of India [38–42]. This choice ensures easy replicability of the study and facilitates cross-regional comparison of findings.

The primary objective of the EAT-10 questionnaire is to identify individuals who are at risk for oro-pharyngeal and oesophageal dysphagia [17]. It comprises ten questions that inquire about the respondents’ day-to-day experiences of swallowing difficulties, using a 5-point Likert scale for rating. Participants are required to assign scores ranging from ‘*0*’ indicating ‘*no problem in swallowing*’ to ‘*4*’ indicating a ‘*severe problem in swallowing*’. As such, each participant’s total EAT-10 score can range from a minimum of 0 to a maximum of 40. A total score of ≥3 is established as the cut-off score indicating self-reported swallowing difficulties [17, 24]. A synthesis of EAT-10 reported the questionnaire to have a pooled sensitivity of 0.89%, pooled specificity of 0.59%, and positive predictive values ranging from 52.40% to 97.14% and negative predictive values ranging from 29.63% to 98.51% [24]. Psychometric properties of EAT-10 in English [17] and Kannada [38] languages are reported to demonstrate good to excellent internal consistency, test–retest reliability, and concurrent validity.

The DHI is a questionnaire that assesses patient-reported swallow outcomes [9]. The primary objective of the DHI is to evaluate physical, functional, and emotional domains associated with swallowing-related QOL [9]. The questionnaire is structured into two domains. The first domain comprises 25 items, including 9 questions each for physical and functional aspects, and 7 questions for the emotional aspect of swallowing. Participants assign a score to each question using a 3-point rating scale, where ‘*0*’ corresponds to ‘*never*’, ‘*2*’ to ‘*sometime*’ and ‘*4*’ to ‘*always*’. The cumulative score for each participant ranges from a minimum of 0 to a maximum of 100. A total score of ≥4 indicates self-reported poor swallowing-related QOL, with higher DHI scores indicating greater negative impact on QOL [44]. In the second domain, participants rate the overall severity of swallowing difficulties using a 7-point equal appearing interval scale. This scale ranges from ‘*1*’ indicating ‘*no difficulty at all*’ through ‘*4*’ indicating ‘*somewhat a problem*’ till ‘*7*’ indicating ‘*the worse problem you could have*’. The English [9] and Kannada [39] DHI have a good internal consistency, test re-test reliability, and concurrent validity.

### Procedure

The participants were provided with the study objectives and the inclusion–exclusion criteria and were recruited after receiving written informed consent. The participants were provided with the following written instructions within the questionnaire. For the EAT-10, participants were provided the following instructions “*Select the response that best suits your day-do-day swallowing experiences. Score of 0 indicates no problem in swallowing and with increase in number the degree of swallowing difficulty increases with 4 suggesting severe swallowing problem*”. As for the DHI, participants were provided the following instructions “*Kindly place a check in the box that best describes your swallowing difficulties*”. These instructions were given to ensure participants’ accurate and consistent completion of the EAT-10 and DHI sections. Participants were given the choice to select either English (Appendix 1) or Kannada (Appendix 2) as their preferred language for survey completion. Assistance in filling out the questionnaires was available to participants only upon request. The survey was conducted in a single session, typically taking participants 15–20 min to complete both questionnaires.

### Data Analysis

The first author of the study compiled all responses, carefully reviewing them to identify any instances of incompleteness, missing information, inaccuracies, or repeated submissions, with the aim of excluding such data from the analysis. This step ensured that only quality data were selected for further statistical analysis. All 361 submissions were examined to assess self-reported swallowing difficulties and swallowing-related QOL among older adults. Participants were categorized as self-reporting swallowing difficulty and reduced swallowing-related QOL if they exhibited challenges while swallowing according to the EAT-10 and DHI criteria, respectively. This decision was guided by a thorough literature search aimed at identifying normative data for both the EAT-10 [17, 24] and DHI [44] questionnaires.

In line with the parent study [17] and meta-analysis [24] of EAT-10, participants with a total score of ≥3 were identified as self-reporting swallowing difficulties. In the present study, we determined the prevalence rate of self-reported swallowing difficulties by calculating the number of participants scoring ≥3 in the EAT-10 relative to the total number of participants who completed the EAT-10 questionnaire and converting the result to a percentage. Similarly, older adults obtaining a total DHI score of ≥4 were identified as self-reporting poor swallowing-related QOL, as outlined in the meta-analysis study on normative values of the DHI [44]. Likewise, we estimated the prevalence of self-reported poor swallowing-related QOL by calculating the number of participants scoring ≥4 in the total DHI relative to the total number who completed the DHI questionnaire and converting the result to a percentage. Further, regarding the overall severity of swallowing difficulties, a rating of ‘*1*’ suggests ‘*normal swallowing-related QOL*’, while ‘*2*’ and ‘*3*’ indicate ‘*mild*’, ‘*4*’ and ‘*5*’ signify ‘*moderate*’ and ‘*6*’ and ‘*7*’ suggest ‘*severe impact on swallowing-related QOL*’, as mentioned in the parent article of DHI [9]. DHI scores across physical, functional, emotional and total DHI were computed under each of the overall severity ratings. To investigate swallowing-related QOL among older adults with and without self-reported swallowing difficulties, we estimated DHI scores for the pass and fail sub-groups of EAT-10.

### Statistical Analysis

All statistical analysis was conducted using Statistical Package for Social Studies version 25 (SPSS Inc, IBM, Chicago). Continuous data from the EAT-10 and DHI questionnaires were summarized through frequency distribution, mean, standard deviation, and minimum–maximum values. An independent *t*-test was run to evaluate potential statistically significant differences in EAT-10 scores between pass and fail groups and gender. Furthermore, a multivariate analysis of variance (MANOVA) test was conducted to explore the statistical significance between pass and fail groups across physical, functional, emotional, and total DHI scores, including overall severity of swallowing difficulties. To check observed differences across specific pairs, Bonferroni post hoc test was conducted. To evaluate potential associations between the pass–fail cohorts of EAT-10 and total DHI, an independent *t*-test was run. The correlation between age and EAT-10 scores for self-reported swallowing difficulties, as well as between age and total DHI scores for swallowing-related QOL, was estimated using the Karl Pearson’s correlation test. Additionally, the correlation between EAT-10 and total DHI was also examined using the Karl Pearson’s correlation test. All statistical tests were conducted with a significance level set at 0.05.

## Results

The overall mean EAT-10 score was 3.34 ± 5.20. Females (3.83 ± 5.64) had higher scores compared to males (2.84 ± 4.68), and this sex difference was statistically significant [*t*(359) = 1.775, *p* = 0.017] at *p* < 0.05. Among older adults, 36.6% (*n* = 132) failed the EAT-10 (8.58 ± 5.47), indicating self-reported swallowing difficulties (Table 1). Independent *t*-test revealed a statistically significant difference between individuals with and without self-reported swallowing difficulties [*t*(359) = −22.566, *p* = 0.000] at *p* < 0.001. Among older adults with self-reported swallowing difficulties, 54.6% (*n* = 72) were females, and 45.4% (*n* = 60) were males. Further, 34.1% (*n* = 123) of older adults reported swallowing difficulties for the inquiry “*Swallowing solids takes extra effort*” followed by 32.7% (*n* = 118) for “*I cough when I eat*” and 29.9% (*n* = 108) for “*When I swallow food sticks in my throat*” (Table 2).

**Table 1:** Results of EAT-10

	*n* (%)	Mean	SD	Range
Pass	229 (63.4)	0.32	0.66	0–2
Fail	132 (36.6)	8.58	5.47	3–27
Total	361 (100)	3.34	5.20	0–27

*SD* standard deviation

**Table 2:** Frequency distribution of EAT-10 responses

Item	Question	0	1	2	3	4
		*n* (%)	*n* (%)	*n* (%)	*n* (%)	*n* (%)
1.	My swallowing problem has caused me to lose weight	316 (87.5)	32 (8.9)	7 (1.9)	4 (1.1)	2 (0.6)
2.	My swallowing problem interferes with my ability to go out for meals	300 (83.1)	44 (12.2)	4 (1.1)	10 (2.8)	3 (0.8)
3.	Swallowing liquids takes extra effort	280 (77.5)	52 (14.4)	21 (5.8)	6 (1.7)	2 (0.6)
4.	Swallowing solids takes extra effort	238 (65.9)	64 (17.7)	32 (8.9)	22 (6.1)	5 (1.4)
5.	Swallowing pills takes extra effort	263 (72.9)	53 (14.7)	37 (10.2)	7 (1.9)	1 (0.3)
6.	Swallowing is painful	327 (90.6)	24 (6.6)	7 (1.9)	2 (0.6)	1 (0.3)
7.	The pleasure of eating is affected by my swallowing	300 (83.1)	39 (10.8)	14 (3.9)	8 (2.2)	0 (0)
8.	When I swallow food sticks in my throat	253 (70.1)	51 (14.1)	48 (13.3)	7 (1.9)	2 (0.6)
9.	I cough when I eat	243 (67.3)	84 (23.3)	26 (7.2)	7 (1.9)	1 (0.3)
10.	Swallowing is stressful	303 (83.9)	35 (9.7)	21 (5.8)	1 (0.3)	1 (0.3)

The mean total DHI was 7.56 ± 12.33, with females scoring higher (8.48 ± 13.70) than males (6.66 ± 10.77). However, the sex differences in total DHI were not statistically significant at *p* > 0.05. 47.4% (*n* = 171) of older adults scored 15.61 ± 14.05 in total DHI (Table 3), which is greater than the normative value ≥4, suggesting swallowing difficulties has a negative impact on their QOL. In older adults self-reporting poor swallowing-related QOL, 52.1% (*n* = 89) were females, and 47.9% (*n* = 82) were males. Older adults self-reported higher scores on the physical scale of the DHI than functional and emotional scales (Table 3). MANOVA test revealed a statistically significant difference between older adults with and without self-reported poor swallowing-related QOL for physical [*F*(1) = 473.712, *p* = 0.000], functional [*F*(1) = 132.748, *p* = 0.000], emotional [*F*(1) = 52.941, *p* = 0.000], and total DHI scores [*F*(1) = 224.733, *p* = 0.000] at *p* < 0.001. Pairwise comparison by Bonferroni post hoc test also revealed a statistically significant difference at *p* < 0.001 (Table 4). Further, 35.7% (*n* = 129) older adults reported swallowing difficulty for the query “*I need to drink fluids to wash food down*”, followed by 29.6% (*n* = 107) “*I feel depressed because I can’t eat what I want*” and for 29.1% (*n* = 105) “*I avoid some foods because of my swallowing problem*” (Table 5).

**Table 3:** Results of DHI

Score	≤3 (Pass)		≥4 (Fail)		Total	
*n* (%)	190 (52.6)		171 (47.4)		361 (100)	
	Mean (SD)	Range	Mean (SD)	Range	Mean (SD)	Range
Physical	0.22 (0.63)	0–2	7.70 (4.69)	2–24	3.76 (4.96)	0–24
Functional	0.06 (0.35)	0–2	5.45 (6.44)	0–30	2.61 (5.18)	0–30
Emotional	0.03 (0.25)	0–2	2.47 (4.61)	0–28	1.19 (3.40)	0–28
Total DHI	0.32 (0.73)	0–2	15.61 (14.05)	4–76	7.56 (12.33)	0–76

*SD* standard deviation

**Table 4:** Post hoc results of DHI between pass-fail groups

	Group		Mean difference	Standard error	Significance
Physical	Pass	Fail	−7.475^*^	0.343	0.000
	Fail	Pass	7.475^*^	0.343	0.000
Functional	Pass	Fail	−5.387^*^	0.468	0.000
	Fail	Pass	5.387^*^	0.468	0.000
Emotional	Pass	Fail	−2.436^*^	0.335	0.000
	Fail	Pass	2.436^*^	0.335	0.000
Total DHI	Pass	Fail	−15.298^*^	1.020	0.000
	Fail	Pass	15.298^*^	1.020	0.000

^*^The mean difference is significant at the 0.05 level

**Table 5:** Frequency distribution of DHI responses

Scale	Question	Never	Sometime	Always
		n (%)	n (%)	n (%)
1P	I cough when I drink liquids	276 (76.5)	82 (22.7)	3 (0.8)
2P	I cough when I eat solid food	286 (79.2)	75 (20.8)	0 (0)
3P	My mouth is dry	268 (74.2)	79 (21.9)	14 (3.9)
4P	I need to drink fluids to wash food down	232 (64.3)	114 (31.6)	15 (4.1)
5P	I’ve lost weight because of my swallowing problem	335 (92.8)	22 (6.1)	4 (1.1)
1F	I avoid some foods because of my swallowing problem	256 (70.9)	88 (24.4)	17 (4.7)
2F	I have changed the way I swallow to make it easier to eat	320 (88.6)	40 (11.1)	1 (0.3)
1E	I’m embarrassed to eat in public	323 (89.5)	36 (10)	2 (0.5)
3F	It takes me longer to eat a meal than it used to	331 (91.7)	29 (8)	1 (0.3)
4F	I eat smaller meals more often due to my swallowing problem	321 (88.9)	32 (8.9)	8 (2.2)
6P	I have to swallow again before food will go down	310 (85.8)	41 (11.4)	10 (2.8)
2E	I feel depressed because I can’t eat what I want	254 (70.4)	45 (12.5)	62 (17.1)
3E	I don’t enjoy eating as much as I used to	301 (83.4)	46 (12.7)	14 (3.9)
5F	I don’t socialize as much due to my swallowing problem	348 (96.4)	8 (2.2)	5 (1.4)
6F	I avoid eating because of my swallowing problem	342 (94.7)	14 (3.9)	5 (1.4)
7F	I eat less because of my swallowing problem	331 (91.7)	23 (6.4)	7 (1.9)
4E	I am nervous because of my swallowing problem	358 (99.2)	2 (0.5)	1 (0.3)
5E	I feel handicapped because of my swallowing problem	330 (91.4)	25 (6.9)	6 (1.7)
6E	I get angry at myself because of my swallowing problem	334 (92.5)	20 (5.6)	7 (1.9)
7P	I choke when I take my medication	339 (93.9)	17 (4.7)	5 (1.4)
7E	I’m afraid that I’ll choke and stop breathing because of my swallowing problem	307 (85)	39 (10.8)	15 (4.2)
8F	I must eat another way (e.g., feeding tube) because of my swallowing problem	346 (95.8)	10 (2.8)	5 (1.4)
9F	I’ve changed my diet due to my swallowing problem	345 (95.6)	13 (3.6)	3 (0.8)
8P	I feel a strangling sensation when I swallow	348 (96.4)	10 (2.8)	3 (0.8)
9P	I cough up food after I swallow	335 (92.8)	23 (6.4)	3 (0.8)

*P* physical domain, *F* functional domain, *E* emotional domain

In the overall severity of swallowing difficulties, 29.1% (*n* = 105) older adults reported their overall difficulties ranging from mild to severe (Table 6). Total DHI scores increased as the overall severity of swallowing difficulties increased. In the subscales, the physical domain of DHI had higher scores compared to the functional and emotional domains in normal and mild overall severity of swallowing difficulties. However, in moderate severity, both physical and functional domains were equally affected, while functional scores were greater than physical domain in the severe category. MANOVA test revealed a statistically significant difference across normal to severe ratings for physical [*F*(3) = 157.771, *p* = 0.000], functional [*F*(3) = 112.850, *p* = 0.000], emotional [*F*(3) = 63.976, *p* = 0.000], and total DHI [*F*(3) = 149.271, *p* = 0.000] at *p* < 0.001. Bonferroni post hoc test also revealed a statistically significant difference at *p* < 0.05 (Table 7).

**Table 6:** Results of DHI based on overall severity of swallowing difficulties

Severity	Normal		Mild		Moderate		Severe	
n (%)	256 (70.9)		82 (22.7)		20 (5.6)		3 (0.8)	
	Mean (SD)	Range	Mean (SD)	Range	Mean (SD)	Range	Mean (SD)	Range
Physical	1.48 (2.53)	0–14	8.41 (4.42)	0–20	11.70 (5.20)	0–20	18.67 (6.11)	12–24
Functional	0.66 (2.08)	0–24	5.73 (5.65)	0–26	11.70 (8.16)	0–30	23.33 (1.15)	22–24
Emotional	0.29 (1.71)	0–24	2.12 (3.40)	0–22	6.80 (7.15)	0–28	14.67 (5.03)	10–20
Total DHI	2.43 (5.29)	0–60	16.27 (11.47)	0–64	30.20 (18.20)	0–76	56.67 (9.24)	46–62

*SD* standard deviation

**Table 7:** Post hoc results of different domains of DHI across overall severity of swallowing difficulties

	Severity		Mean difference	Standard error	Significance
Physical	Normal	Mild	−6.938^*^	0.414	0.000
		Moderate	−10.223^*^	0.758	0.000
		Severe	−17.190^*^	1.895	0.000
	Mild	Normal	6.938^*^	0.414	0.000
		Moderate	−3.285^*^	0.814	0.000
		Severe	−10.252^*^	1.918	0.000
	Moderate	Normal	10.223^*^	0.758	0.000
		Mild	3.285^*^	0.814	0.000
		Severe	−6.967^*^	2.020	0.004
	Severe	Normal	17.190^*^	1.895	0.000
		Mild	10.252^*^	1.918	0.000
		Moderate	6.967^*^	2.020	0.004
Functional	Normal	Mild	−5.068^*^	0.473	0.000
		Moderate	−11.036^*^	0.866	0.000
		Severe	−22.669^*^	2.166	0.000
	Mild	Normal	5.068^*^	0.473	0.000
		Moderate	−5.968^*^	0.930	0.000
		Severe	−17.602^*^	2.192	0.000
	Moderate	Normal	11.036^*^	0.866	0.000
		Mild	5.968^*^	0.930	0.000
		Severe	−11.633^*^	2.309	0.000
	Severe	Normal	22.669^*^	2.166	0.000
		Mild	17.602^*^	2.192	0.000
		Moderate	11.633^*^	2.309	0.000
Emotional	Normal	Mild	−1.833^*^	0.349	0.000
		Moderate	−6.511^*^	0.639	0.000
		Severe	−14.378^*^	1.598	0.000
	Mild	Normal	1.833^*^	0.349	0.000
		Moderate	−4.678^*^	0.686	0.000
		Severe	−12.545^*^	1.618	0.000
	Moderate	Normal	6.511^*^	0.639	0.000
		Mild	4.678^*^	0.686	0.000
		Severe	−7.867^*^	1.704	0.000
	Severe	Normal	14.378^*^	1.598	0.000
		Mild	12.545^*^	1.618	0.000
		Moderate	7.867^*^	1.704	0.000
Total DHI	Normal	Mild	−13.839^*^	1.046	0.000
		Moderate	−27.770^*^	1.914	0.000
		Severe	−54.237^*^	4.788	0.000
	Mild	Normal	13.839^*^	1.046	0.000
		Moderate	−13.932^*^	2.056	0.000
		Severe	−40.398^*^	4.847	0.000
	Moderate	Normal	27.770^*^	1.914	0.000
		Mild	13.932^*^	2.056	0.000
		Severe	−26.467^*^	5.105	0.000
	Severe	Normal	54.237^*^	4.788	0.000
		Mild	40.398^*^	4.847	0.000
		Moderate	26.467^*^	5.105	0.000

^*^The mean difference is significant at the 0.05 level

**Fig. 1:**
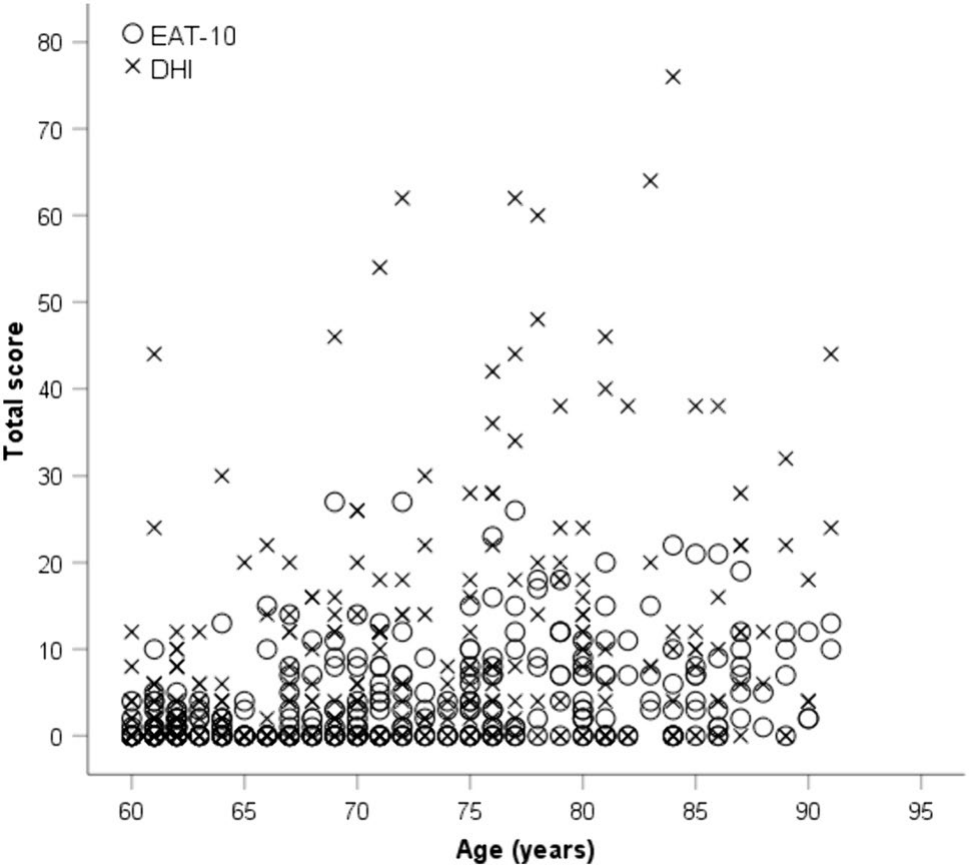
Scatter plot of self-reported scores against age

A comparison between EAT-10 and total DHI revealed that older adults who passed the EAT-10 (0.32 ± 0.66) had total DHI scores within normal limits (1.86 ± 3.31). However, older adults who failed the EAT-10 (8.58 ± 5.47) had total DHI scores greater than normative data (17.45 ± 15.59), suggesting that their swallowing difficulties had a negative impact on their QOL. A Karl Pearsons correlation coefficient value of *r* = 0.86 indicated a strong positive correlation between EAT-10 and total DHI scores at *p* = 0.000. When comparing age with EAT-10 and total DHI scores (Fig. 1), Karl Pearsons correlation coefficient values of 0.41 and 0.34 were obtained, suggesting moderate and weak positive correlation, respectively.

## Discussion

The present study estimated self-reported swallowing difficulties using the EAT-10 and assessed swallowing-related QOL using DHI questionnaire among community-dwelling older adults in India. The findings of this study add to existing literature, confirming that age significantly impacts swallowing, with a substantial proportion of older adults self-reporting swallowing difficulties [10–16, 18–20].

Table 8 presents the prevalence of self-reported swallowing difficulties among community dwelling-older adults from various countries, as estimated using EAT-10 questionnaire. As illustrated in Table 8, the prevalence rates range from 5.7% to 53.8% across different studies, encompassing a minimum of 443 community-dwelling older adults to maximum of 4403 participants. Additionally, the mean EAT-10 scores reported in these studies varied between 0.72 and 5.8. Notably, the results of our study are consistent with the literature, as we observed a comparable estimated prevalence of 36.6% within our cohort of 361 older adults.

**Table 8:** Study details of prevalence of self-reported swallowing difficulties in community-dwelling older adults across countries estimated by EAT-10

Country	Author details	Age range	*n*	Data collection	Cut-off	Mean ± SD	Prevalence	Top 3 complaints (%)
Brazil	Dantas et al. [12]	20–84	443	NDA	≥3	0.72 ± 1.85	9.5%	1. Q5 (17.2) 2. Q8 (9.5) 3. Q9 (7.7)
China	Zhang et al. [16]	>65	4403	Interview	≥3	NDA	5.7%	NDA
Denmark	Kertscher et al. [15]	>18	2600	Telephonic	≥3	NDA	9.8% (61–75 years) 21.9% (>76 years)	1. Q5 (33) 2. Q8 (19) 3. Q9 (13)
					≥2	NDA	12.4% (61–75 years) 26.2% (>76 years)	1. Q5 (48) 2. Q8 (24) 3. Q9 (26)
Japan	Igarashi et al. [13]	>65	510	Post-mails	≥3	2 ± 3.6	25.1% (independent community-dwellers)	1. Q9 (NDA) 2. NDA 3. NDA
			886			5.8 ± 6.9	53.8% (long-term care insurance beneficiaries)	NDA
New Zealand	Jardine et al. [14]	65–96	1020	Email	˃3	2.15 ± 4.3	22.1%	NDA
Thailand	Chaleekrua et al. [10]	>60	874	Interview	≥3	NDA	11%	1. Q8 (12.9) 2. Q3 (11.0) 3. Q9 (7.7)
USA	Kurosu et al. [28]	>60	476	Interview	≥3	NDA	20.4%	NDA

*n* sample size, *NDA* no details available, *SD* standard deviation

Furthermore, among the seven studies listed in Table 6, six of them utilised a cut-off score of ≥3 on the EAT-10 questionnaire to identify individuals with self-reported swallowing difficulties. One study, however, adopted a cut-off score of ≥2 [15]. In their study, Kertscher et al. [15] reported a prevalence rate of self-reported swallowing difficulties of 9.8–21.9% for a cut-off ≥3. This estimate increased to 12.4–26.2% when the EAT-10 cut-off was reduced to ≥2. It is important to note that this variation in the cut-off score between ≥2 and ≥3 for the EAT-10 is widely discussed in the literature, a point emphasized in the works of Rofes et al. [45]. Their study reported that lowering the cut-off scores to 2 increases the sensitivity without affecting specificity rates, ultimately reducing the false negative rate. However, it is worth mentioning that a recent evidence synthesis study recommended a cut-off score of ≥3 for the EAT-10 questionnaire, as it offers better diagnostic accuracy in identifying individuals at risk for swallowing difficulties [24]. This recommendation guided our choice to follow the ≥3 cut-off score to distinguish community-dwelling older adults with self-reported swallowing difficulties from those without.

In contrast to the wealth of information available regarding the EAT-10 questionnaire, there is a paucity of information regarding the use of DHI to assess the status of swallowing-related QOL among older adults. This point is partly supported by the five studies included in the systematic review and metanalysis, which aimed to establish normative data for DHI [44]. Further, our literature search yielded only two studies that assessed swallowing-related QOL using DHI in older adult populations [18, 46]. In the study conducted by Mulheren et al. [18], it was found that 60% (*n* = 12) of the older adults self-reported a poor swallowing-related QOL, with a total DHI score averaging 7.25 ± 10.75. However, it is important to exercise caution when generalizing these findings, given the relatively small sample size of 32 older adults and the wide age range, spanning from 62 to 91 years. Another study by Hazelwood et al. [46] reported that older adults experienced a moderate impact on their swallowing-related QOL. It is worth noting that a direct comparison between our study and Hazelwood et al. [46] is not feasible, as their study also included younger and middle-aged adults, ranging from 25 years to older adults aged up to 95 years. Therefore, further research is warranted to assess swallowing-related QOL among community-dwelling older adults using the DHI questionnaire to draw conclusions.

Older adults in our study reported the highest swallowing difficulties for specific symptoms, including difficulty swallowing solids, cough while swallowing, and food sticking in throat, as indicated by their responses on the EAT-10 questionnaire. These findings align with previous literature that older adults frequently experience coughing and food sticks in throat [10, 12, 13, 15]. Interestingly, the reports of difficulty swallowing solids are a novel observation among older adults, not previously reported in prevalence studies that have utilized EAT-10 questionnaire. However, objective evaluations of swallowing, such as the Test of Mastication and Swallowing Solids, have reported a functional decline in swallowing solids with increase in age [47, 48]. Further investigation is warranted to explore the underlying mechanisms for addressing the emerging issue of difficulty swallowing solids in older adults, while also continuing to examine the broader spectrum of swallowing difficulties and their impact on aging populations.

In the total DHI domain, our results suggest that older adults scored the highest on the physical scale, indicating a significant impact on their physical well-being due to swallowing difficulties. These findings are consistent with the study conducted by Mulheren et al. [18], where older adults scored 4.63 ± 5.62 on the physical scale, 1.5 ± 3.45 on functional and 1.13 ± 2.78 on the emotional scale. Furthermore, when examining the top three highest reported swallowing difficulties in each scale, it becomes evident that these difficulties are spread equally (one question each) across the physical (drink liquids to wash food down), functional (avoid some food because of swallowing difficulties), and emotional (feel depressed for not able to eat what I want) scales. This highlights the comprehensive impact of swallowing difficulties on an older adult’s overall swallowing-related QOL, encompassing physical, emotional, and functional aspects.

In our study, the mean scores for participants failing the EAT-10 and DHI were relatively low. Upon closer examination of the data, it was observed that the difference in the range of swallowing difficulties was also minimal. This suggests that swallowing difficulties in older adults of the study may not be severe. This notion is further highlighted by the results indicating that the overall severity of swallowing difficulties was predominantly rated as ‘*normal*’ and ‘*mild*’. In addition, females gave higher self-ratings compared to males. These results support previous research indicating that older female adults are more likely to report higher levels of swallowing difficulties than males [2, 12, 25, 49]. Similar to age being an independent predictor of self-reported swallowing difficulties, female older adults are reported to be 20% more likely to self-report swallowing difficulties than age-matched males [2]. Furthermore, Dantas et al. [12] study reported that 11.7% of females self-reported swallowing difficulties with EAT-10 scores ranging 0–20, in contrast to 5.9% of males who had EAT-10 scores of 0–8 at *p* < 0.01. However, when swallowing was longitudinally monitored at two time points, a significant association was reported between total scores of the Sydney Swallow Questionnaire with age and sex in 2009, which changed to no significant association with age and sex in 2012 [19]. Differences in the results of age and sex cohorts between the study using EAT-10 [12] and Sydney Swallow Questionnaire [19] can be attributed to the differing designs of the two questionnaires [20]. Overall, these findings emphasize the need for healthcare professionals to consider sex-specific factors when evaluating and addressing swallowing difficulties in older adults. A more thorough approach that recognizes sex-related differences in symptoms and outcomes can enhance dysphagia screening.

This study is the first to use EAT-10 and DHI questionnaires to assess self-reported swallowing difficulties and swallowing-related QOL in community-dwelling older adults. We obtained a strong positive correlation between EAT-10 and DHI scores at *p* < 0.001. These findings are consistent with those of Hazelwood et al. [46], who also reported a strong positive correlation (*r* = 0.817) between EAT-10 and DHI scores. However, Hazelwood et al. [46] cautioned against using both the EAT-10 and DHI due to the potential redundancy in measurement tools. This observation raises an interesting point because the EAT-10 primarily serves as a screening tool for identifying swallowing difficulties, while the DHI is designed to assess swallow outcomes related to swallowing-related QOL. Further research and discussion are warranted to explore the potential overlap or complementarity of these two tools in assessing swallowing difficulties and their impact on the QOL among older adults. Nonetheless, we anticipate a shift in the trend of utilizing DHI from solely a patient-reported outcome questionnaire to a self-reporting tool for dysphagia symptoms, especially with the publication of normative data [44]. To our knowledge, this is the first study to use DHI to determine the prevalence of self-reported swallowing-related QOL among community-dwelling older adults.

### Limitations

The findings of present study come with a few limitations that should be acknowledged. Firstly, the older adults in the study did not undergo formal evaluations of health fitness, including assessments of nutrition status, frailty, education levels, and socio-economic status. Data collection relied on self-reports of good health among older adults. Not controlling for such factors may have contributed to the prevalence rates reported in the study, potentially overestimating the true prevalence of self-reported swallowing difficulties. Secondly, it’s important to note that the prevalence of self-reported swallowing difficulties reported in this study is specific to community-dwelling older adults. Therefore, caution should be exercised when attempting to generalize these prevalence estimates to older adults residing in institutional settings such as nursing facilities or old-age homes. Thirdly, the participants in the present study were exclusively urban-dwelling older adults. This could introduce a potential bias in the findings, limiting their applicability to older adults in rural areas or different geographical settings. Lastly, the study did not include formal objective tests of swallowing function, such as the Water Swallowing Test or the Test of Mastication and Swallowing Solids. As mentioned in previous literature [49, 50], it is possible that some older adults who did not self-report swallowing difficulties might still have functional swallowing impairments. To address this limitation, future research could consider incorporating objective swallowing tests. Additionally, using open-ended questions like “*Explain your swallowing difficulties*” as done in another study [49], could capture personal experiences of swallowing difficulties that may not be captured by standardized questionnaires like the EAT-10 or DHI.

## Conclusion

In conclusion, our study reveals that 36.6% of community-dwelling older adults in India self-report swallowing difficulties, while 47.4% experience a diminished swallowing-related QOL. This information underscores the importance of Speech-Language Pathologists actively implementing more screening programs to identify older adults at risk of age-related swallowing difficulties. Furthermore, these findings can serve as a valuable resource for patient education. Policymakers can leverage the results of this study to inform policies and healthcare practices specific to India. In the future, studies should supplement self-reports with objective tests of swallowing to determine the presence or absence of swallowing difficulties in older adults. It is also essential to evaluate swallowing capacities of older adults residing in nursing homes and old age homes. Lastly, it would be of interest to compare the reports of swallowing difficulties provided by family members and/or caregivers with self-reports of older adults.

## Supplementary Information

Below is the link to the electronic supplementary material.

Supplementary file1 (DOC 176 KB)

## Data Availability

The datasets generated during and/or analysed during the current study are available from the corresponding author on reasonable request.
