# Supplementary material for: Prevalence of Self-Reported Swallowing Difficulties and Swallowing-Related Quality of Life Among Community-Dwelling Older Adults in India
Source: Dysphagia. 2024 Apr 18;39(6):1144–55. doi: 10.1007/s00455-024-10696-0 (PMC11607026; doi:10.1007/s00455-024-10696-0)
Supplement: Supplementary file 1 — Supplementary file1 (DOC 176 KB) [file 455_2024_10696_MOESM1_ESM.doc]

# Appendix 1

**Survey on self-reported swallowing difficulty among community-dwelling older adults**

**Section 1: Informed consent**

You are being invited to take part in a research study conducted by Mr. Thejaswi Dodderi, pursuing Doctor of Philosophy (PhD) at the Department of Audiology and Speech Language Pathology, Kasturba Medical College, Mangalore. The work is carried out under the guidance of Dr. Radish Kumar Balasubramanium (Professor, Speech Language Pathology). Before you decide whether or not to participate, it is important for you to understand why the research is being done and what it will involve. Please take time to read the following information carefully.

**Why is this study being done?**

The purpose of this study is to measure the self-reported swallowing difficulty and swallowing related quality of life among old adults.

**What will happen if I take part in this study?**

The researcher will collect information about you, your personal information such as your name, date of birth, address, and phone number. All this information will be stored at Kasturba Medical College (KMC), Attavara. If you agree to participate in this research, you are giving permission to use your health information for research. However, your information will only be used by KMC for research purposes only.

**Are there potential benefits to taking part in the study?**

The participants taking part in the study will be assessed for swallowing difficulty using two checklists in English or Kannada language. Swallowing difficulties identified through this survey will be informed by telephone call to the individual by the researcher. These individuals are eligible to receive dysphagia therapy at the Department of Audiology and Speech Language Pathology, Kasturba Medical College, Attavar.

**What are the risks of the study?**

There are no physical risks associated with this study.

**What are the costs?**

There are no additional costs to you associated with participating in this research study.

**What about confidentiality?**

Information from this study including your name, age, and health details will be reviewed only by authorized personnel or representatives, ethics committee or regulatory bodies who will be responsible for making this research and drawing proper inferences and conclusions. Information and results from the study may be presented at meetings or published in journals without including your name and other personal identifications. The retrieved data will be destroyed after meeting the purpose of the study.

**What are my rights?**

Your participation in this research study is voluntary. You may choose not to be in the study. If you agree to be in the study, you may withdraw from the study at any time. If you withdraw from the study, no new data about you will be collected for research purposes. Your decision not to participate or to withdraw from the study will not involve any penalty or loss of benefits. You will continue to receive your usual medical care from the hospital as well as department whether or not you decide to participate in this study. After you had a chance to read this information sheet and decided whether you want to participate, please let the researcher know what you have decided. You are required to sign a consent form to participate in this research, but you must let the researcher know whether or not you wish to participate. If you have any questions regarding the research study or the information sheet, please ask the researcher.

You will be given a copy of this information sheet to take home with you. Please reach out to the researcher for any queries or doubts on 09108788160.

I have read the informed consent and agree to participate in the survey:

- Yes, I consent to enroll in the survey *(automatically redirects to Section 2)*
- No, I don’t consent to enroll in the survey *(terminates the survey)*

**Section 2: Demographic and medical data**

Instructions: Fill the answers carefully for each question

1. Name

*Paragraph box*

1. Age (in years)

*Paragraph box*

1. Sex

*Male*

*Female*

*Others*

1. Have you undergone surgery to the head, neck, or other body parts?

*Yes*

*No*

1. If yes, mention the details. If no, leave it blank

*Paragraph box*

1. Have you had stroke in the last 5 years?

*Yes*

*No*

1. If yes, mention the details. If no, leave it blank

*Paragraph box*

1. Did the doctor tell you have hypertension, diabetes, cancer, chronic lung disease, heart attack, congestive heart failure, asthma, arthritis, stroke, and kidney disease?

*Yes*

*No*

1. If yes, mention the details. If no, leave it blank

*Paragraph box*

1. Mention other health issues you have (Leave it blank if you are healthy)

*Paragraph box*

1. How much do you currently weigh? (in kgs)

*Paragraph box*

1. How tall are you? (in cms or feet)

*Paragraph box*

**Section 3: Eating Assessment Tool-10**

Instructions:

- Circle the appropriate response that best suits your day-to-day swallowing experience
- Score of 0 indicates no problem in swallowing and with increase in number the degree of swallowing difficulty increases with 4 suggesting severe swallowing problem

1. My swallowing problem has caused me to lose weight

| *0* | *1* | *2* | *3* | *4* |
| --- | --- | --- | --- | --- |
| *No problem* |  |  |  | *Severe problem* |

1. My swallowing problem interferes with my ability to go out for meals

| *0* | *1* | *2* | *3* | *4* |
| --- | --- | --- | --- | --- |
| *No problem* |  |  |  | *Severe problem* |

1. Swallowing liquids takes extra effort

| *0* | *1* | *2* | *3* | *4* |
| --- | --- | --- | --- | --- |
| *No problem* |  |  |  | *Severe problem* |

1. Swallowing solids takes extra effort

| *0* | *1* | *2* | *3* | *4* |
| --- | --- | --- | --- | --- |
| *No problem* |  |  |  | *Severe problem* |

1. Swallowing pills takes extra effort

| *0* | *1* | *2* | *3* | *4* |
| --- | --- | --- | --- | --- |
| *No problem* |  |  |  | *Severe problem* |

1. Swallowing is painful

| *0* | *1* | *2* | *3* | *4* |
| --- | --- | --- | --- | --- |
| *No problem* |  |  |  | *Severe problem* |

1. The pleasure of eating is affected by my swallowing

| *0* | *1* | *2* | *3* | *4* |
| --- | --- | --- | --- | --- |
| *No problem* |  |  |  | *Severe problem* |

1. When I swallow food sticks in my throat

| *0* | *1* | *2* | *3* | *4* |
| --- | --- | --- | --- | --- |
| *No problem* |  |  |  | *Severe problem* |

1. I cough when I eat

| *0* | *1* | *2* | *3* | *4* |
| --- | --- | --- | --- | --- |
| *No problem* |  |  |  | *Severe problem* |

1. Swallowing is stressful

| *0* | *1* | *2* | *3* | *4* |
| --- | --- | --- | --- | --- |
| *No problem* |  |  |  | *Severe problem* |

**Section 4: Dysphagia Handicap Index**

Instructions:

- Please place a check in the box that describes your swallowing difficulty

1. I cough when I drink liquids

| *Never* | *Sometime* | *Always* |
| --- | --- | --- |

1. I cough when I eat solid food

| *Never* | *Sometime* | *Always* |
| --- | --- | --- |

1. My mouth is dry

| *Never* | *Sometime* | *Always* |
| --- | --- | --- |

1. I need to drink fluids to wash food down

| *Never* | *Sometime* | *Always* |
| --- | --- | --- |

1. I’ve lost weight because of my swallowing problem

| *Never* | *Sometime* | *Always* |
| --- | --- | --- |

1. I avoid some foods because of my swallowing

| *Never* | *Sometime* | *Always* |
| --- | --- | --- |

1. I have changed the way I swallow to make it easier to eat

| *Never* | *Sometime* | *Always* |
| --- | --- | --- |

1. I’m embarrassed to eat in public

| *Never* | *Sometime* | *Always* |
| --- | --- | --- |

1. It takes me longer to eat a meal than it used to

| *Never* | *Sometime* | *Always* |
| --- | --- | --- |

1. I eat smaller meals more often due to my swallowing problem

| *Never* | *Sometime* | *Always* |
| --- | --- | --- |

1. I have to swallow again before food will go down

| *Never* | *Sometime* | *Always* |
| --- | --- | --- |

1. I feel depressed because I can’t eat what I want

| *Never* | *Sometime* | *Always* |
| --- | --- | --- |

1. I don’t enjoy eating as much as I used to

| *Never* | *Sometime* | *Always* |
| --- | --- | --- |

1. I don’t socialize as much due to my swallowing problem

| *Never* | *Sometime* | *Always* |
| --- | --- | --- |

1. I avoid eating because of my swallowing problem

| *Never* | *Sometime* | *Always* |
| --- | --- | --- |

1. I eat less because of my swallowing problem

| *Never* | *Sometime* | *Always* |
| --- | --- | --- |

1. I am nervous because of my swallowing problem

| *Never* | *Sometime* | *Always* |
| --- | --- | --- |

1. I feel handicapped because of my swallowing problem

| *Never* | *Sometime* | *Always* |
| --- | --- | --- |

1. I get angry at myself because of my swallowing problem

| *Never* | *Sometime* | *Always* |
| --- | --- | --- |

1. I choke when I take my medication

| *Never* | *Sometime* | *Always* |
| --- | --- | --- |

1. I’m afraid that I’ll choke and stop breathing because of my swallowing problem

| *Never* | *Sometime* | *Always* |
| --- | --- | --- |

1. I must eat another way (e.g., feeding tube) because of my swallowing problem

| *Never* | *Sometime* | *Always* |
| --- | --- | --- |

1. I’ve changed my diet due to my swallowing problem

| *Never* | *Sometime* | *Always* |
| --- | --- | --- |

1. I feel a strangling sensation when I swallow

| *Never* | *Sometime* | *Always* |
| --- | --- | --- |

1. I cough up food after I swallow

| *Never* | *Sometime* | *Always* |
| --- | --- | --- |

1. Please circle the number that matches the severity of your swallowing difficulty (1 = no difficulty at all; 4 = somewhat of a problem; 7 = the worse problem you could have)

| 1 | 2 | 3 | 4 | 5 | 6 | 7 |
| --- | --- | --- | --- | --- | --- | --- |
| *Normal* |  | *Moderate problem* | | | *Severe problem* | |

# Appendix 2

**ವಯಸ್ಸಾದ ವ್ಯಕ್ತಿಗಲ್ಲಿ ಸ್ವಯಂ-ವರದಿ ಮಾಡಿದ ನುಂಗುವ ತೊಂದರೆಯ ಮೇಲೆ ಸಮೀಕ್ಷೆ**

**ವಿಭಾಗ 1: ತಿಳುವಳಿಕೆಯುಳ್ಳ ಒಪ್ಪಿಗೆ**

ಮಂಗಳೂರಿನ ಅತ್ತವರ ಕಸ್ತೂರ್ಬಾ ವೈದ್ಯಕೀಯ ಕಾಲೇಜಿನಲ್ಲಿ ಡಾಕ್ಟರ್ ಆಫ್ ಫಿಲಾಸಫಿ (ಪಿಎಚ್‌ಡಿ) ವ್ಯಾಸಂಗ ಮಾಡುತ್ತಿರುವ ಶ್ರೀ ತೇಜಸ್ವಿ ಡಿ ನಡೆಸಿದ ಸಂಶೋಧನಾ ಅಧ್ಯಯನದಲ್ಲಿ ಭಾಗವಹಿಸಲು ನಿಮ್ಮನ್ನು ಆಹ್ವಾನಿಸಲಾಗುತ್ತಿದೆ. ಡಾ. ರಾದೀಶ್ ಕುಮಾರ್ ಬಿ (ಪ್ರೊಫೆಸರ್, ಸ್ಪೀಚ್ ಲ್ಯಾಂಗ್ವೇಜ್ ಪ್ಯಾಥಾಲಜಿ) ಅವರ ಮಾರ್ಗದರ್ಶನದಲ್ಲಿ ಕೆಲಸವನ್ನು ಕೈಗೊಳ್ಳಲಾಗುತ್ತದೆ. ಭಾಗವಹಿಸಬೇಕೆ ಅಥವಾ ಬೇಡವೇ ಎಂಬುದನ್ನು ನೀವು ನಿರ್ಧರಿಸುವ ಮೊದಲು, ಸಂಶೋಧನೆ ಏಕೆ ನಡೆಯುತ್ತಿದೆ ಮತ್ತು ಅದು ಏನನ್ನು ಒಳಗೊಂಡಿರುತ್ತದೆ ಎಂಬುದನ್ನು ನೀವು ಅರ್ಥಮಾಡಿಕೊಳ್ಳುವುದು ಬಹಳ ಮುಖ್ಯ. ದಯವಿಟ್ಟು ಈ ಕೆಳಗಿನ ಮಾಹಿತಿಯನ್ನು ಎಚ್ಚರಿಕೆಯಿಂದ ಓದಲು ಸಮಯ ತೆಗೆದುಕೊಳ್ಳಿ. ನೀವು ಆಯ್ಕೆ ಮಾನದಂಡಗಳನ್ನು ಪೂರೈಸುತ್ತಿರುವುದರಿಂದ ಈ ಸಂಶೋಧನಾ ಅಧ್ಯಯನದಲ್ಲಿ ಭಾಗವಹಿಸಲು ನಿಮ್ಮನ್ನು ಕೇಳಲಾಗುತ್ತದೆ.

**ಈ ಅಧ್ಯಯನವನ್ನು ಏಕೆ ಮಾಡಲಾಗುತ್ತಿದೆ?**

ಈ ಅಧ್ಯಯನದ ಉದ್ದೇಶವು ವಯಸ್ಸಾದ ವ್ಯಕ್ತಿಗಲ್ಲಿ ಸ್ವಯಂ-ವರದಿ ಮಾಡಿದ ನುಂಗುವ ತೊಂದರೆ ಮತ್ತು ನುಂಗಲು ಸಂಬಂಧಿಸಿದ ಜೀವನದ ಗುಣಮಟ್ಟವನ್ನು ಅಳೆಯುವುದು.

**ನಾನು ಈ ಅಧ್ಯಯನದಲ್ಲಿ ಭಾಗವಹಿಸಿದರೆ ಏನಾಗುತ್ತದೆ?**

ಸಂಶೋಧಕರು ನಿಮ್ಮ ಬಗ್ಗೆ, ನಿಮ್ಮ ಹೆಸರು, ವಯಸ್ಸು ಮತ್ತು ಆರೋಗ್ಯ ಮಾಹಿತಿಯನ್ನು ಸಂಗ್ರಹಿಸುತ್ತಾರೆ. ಈ ಎಲ್ಲ ಮಾಹಿತಿಯನ್ನು ಅತ್ತವರ ಕಸ್ತೂರ್ಬಾ ವೈದ್ಯಕೀಯ ಕಾಲೇಜಿನಲ್ಲಿ (ಕೆಎಂಸಿ) ಸಂಗ್ರಹಿಸಲಾಗುವುದು. ಈ ಸಂಶೋಧನೆಯಲ್ಲಿ ಭಾಗವಹಿಸಲು ನೀವು ಒಪ್ಪಿದರೆ, ನಿಮ್ಮ ಆರೋಗ್ಯ ಮಾಹಿತಿಯನ್ನು ಸಂಶೋಧನೆಗೆ ಬಳಸಲು ನೀವು ಅನುಮತಿ ನೀಡುತ್ತಿರುವಿರಿ. ಆದಾಗ್ಯೂ, ನಿಮ್ಮ ಮಾಹಿತಿಯನ್ನು ಸಂಶೋಧನಾ ಉದ್ದೇಶಗಳಿಗಾಗಿ ಮಾತ್ರ ಕೆಎಂಸಿ ಬಳಸುತ್ತದೆ.

**ಅಧ್ಯಯನದಲ್ಲಿ ಪಾಲ್ಗೊಳ್ಳುವುದರಿಂದ ಸಂಭಾವ್ಯ ಪ್ರಯೋಜನಗಳಿವೆಯೇ?**

ಈ ಅಧ್ಯಯನದಲ್ಲಿ ಭಾಗವಹಿಸುವವರನ್ನು ಎರಡು ಪರಿಶೀಲನಾಪಟ್ಟಿಯ ಮುಖಾಂತರ ನುಂಗುವ ಸಮಸ್ಯೆಯನ್ನು ಹಾಗು ದೌರ್ಬಲ್ಯವನ್ನು ಪತ್ತೆಹಚ್ಚಲಾಗುತ್ತದೆ. ನುಂಗುವ ತೊಂದರೆ ಕಂಡುಬಂದಲ್ಲಿ, ಇದನ್ನು ಭಾಗವಹಿಸುವವರಗಮನಕ್ಕೆ ತರಲಾಗುತ್ತದೆ. ಅಷ್ಟರಲ್ಲಿ, ವಿವರವಾದ ಮೌಲ್ಯಮಾಪನಕ್ಕಾಗಿ ಅತ್ತಾವರದ ಕಸ್ತೂರ್ಬಾ ವೈದ್ಯಕೀಯ ಕಾಲೇಜಿನ ಆಡಿಯಾಲಜಿ ಮತ್ತು ಸ್ಪೀಚ್ ಭಾಷಾ ರೋಗಶಾಸ್ತ್ರ ಇಲಾಖೆಗೆ ಸೂಕ್ತವಾದ ಉಲ್ಲೇಖವನ್ನು ನೀಡಲಾಗುವುದು ಮತ್ತು ಪರಿಹಾರ ಕ್ರಮಗಳು ತನಿಖಾಧಿಕಾರಿಯ ಜವಾಬ್ದಾರಿಯಾಗಿದೆ.

**ಅಧ್ಯಯನದ ಅಪಾಯಗಳು ಯಾವುವು?**

ಈ ಅಧ್ಯಯನಕ್ಕೆ ಸಂಬಂಧಿಸಿದ ಯಾವುದೇ ದೈಹಿಕ ಅಪಾಯಗಳಿಲ್ಲ.

**ವೆಚ್ಚಗಳು ಯಾವುವು?**

ಈ ಸಂಶೋಧನಾ ಅಧ್ಯಯನದಲ್ಲಿ ಭಾಗವಹಿಸುವುದರೊಂದಿಗೆ ನಿಮಗೆ ಯಾವುದೇ ಹೆಚ್ಚುವರಿ ವೆಚ್ಚಗಳಿಲ್ಲ.

**ಗೌಪ್ಯತೆಯ ಬಗ್ಗೆ ಏನು?**

ನಿಮ್ಮ ಹೆಸರು, ಹುಟ್ಟಿದ ದಿನಾಂಕ, ವಿಳಾಸ, ಅಧ್ಯಯನದ ಫಲಿತಾಂಶಗಳನ್ನು ಒಳಗೊಂಡಂತೆ ಈ ಅಧ್ಯಯನದ ಮಾಹಿತಿಯನ್ನು ಅಧಿಕೃತ ಸಿಬ್ಬಂದಿ ಅಥವಾ ಪ್ರತಿನಿಧಿಗಳು, ನೈತಿಕ ಸಮಿತಿ ಅಥವಾ ನಿಯಂತ್ರಕ ಸಂಸ್ಥೆಗಳು ಮಾತ್ರ ಪರಿಶೀಲಿಸುತ್ತವೆ, ಅವರು ಈ ಸಂಶೋಧನೆ ಮಾಡಲು ಮತ್ತು ಸರಿಯಾದ ನಿರ್ಣಯಗಳು ಮತ್ತು ತೀರ್ಮಾನಗಳನ್ನು ತೆಗೆದುಕೊಳ್ಳುವ ಜವಾಬ್ದಾರಿಯನ್ನು ಹೊಂದಿರುತ್ತಾರೆ. ನಿಮ್ಮ ಮಾಹಿತಿ ಮತ್ತು ಇತರ ವೈಯಕ್ತಿಕ ಗುರುತುಗಳನ್ನು ಸೇರಿಸದೆಯೇ ಅಧ್ಯಯನದ ಮಾಹಿತಿ ಮತ್ತು ಫಲಿತಾಂಶಗಳನ್ನು ಸಭೆಗಳಲ್ಲಿ ಪ್ರಸ್ತುತಪಡಿಸಬಹುದು ಅಥವಾ ನಿಯತಕಾಲಿಕಗಳಲ್ಲಿ ಪ್ರಕಟಿಸಬಹುದು. ಅಧ್ಯಯನ ಮುಗಿದ ನಂತರ ಸಂಗ್ರಹಿಸಿದ ಮಾಹಿತಿಯನ್ನು ನಾಶಮಾಡಲಾಗುವುದು.

**ನನ್ನ ಹಕ್ಕುಗಳು ಯಾವುವು?**

ಈ ಸಂಶೋಧನಾ ಅಧ್ಯಯನದಲ್ಲಿ ನಿಮ್ಮ ಭಾಗವಹಿಸುವಿಕೆ ಸ್ವಯಂಪ್ರೇರಿತವಾಗಿದೆ. ನೀವು ಅಧ್ಯಯನದಲ್ಲಿ ಇರಬಾರದು ಎಂದು ಆಯ್ಕೆ ಮಾಡಬಹುದು. ನೀವು ಅಧ್ಯಯನದಲ್ಲಿರಲು ಒಪ್ಪಿದರೆ, ನೀವು ಯಾವುದೇ ಸಮಯದಲ್ಲಿ ಅಧ್ಯಯನದಿಂದ ಹಿಂದೆ ಸರಿಯಬಹುದು. ನೀವು ಅಧ್ಯಯನದಿಂದ ಹಿಂದೆ ಸರಿದರೆ, ಸಂಶೋಧನಾ ಉದ್ದೇಶಗಳಿಗಾಗಿ ನಿಮ್ಮ ಬಗ್ಗೆ ಯಾವುದೇ ಹೊಸ ಡೇಟಾವನ್ನು ಸಂಗ್ರಹಿಸಲಾಗುವುದಿಲ್ಲ. ಭಾಗವಹಿಸದಿರಲು ಅಥವಾ ಅಧ್ಯಯನದಿಂದ ಹಿಂದೆ ಸರಿಯುವ ನಿಮ್ಮ ನಿರ್ಧಾರವು ಯಾವುದೇ ದಂಡ ಅಥವಾ ಪ್ರಯೋಜನಗಳ ನಷ್ಟವನ್ನು ಒಳಗೊಂಡಿರುವುದಿಲ್ಲ. ಈ ಅಧ್ಯಯನದಲ್ಲಿ ಭಾಗವಹಿಸಲು ನೀವು ನಿರ್ಧರಿಸುತ್ತೀರೋ ಇಲ್ಲವೋ ಎಂದು ಆಸ್ಪತ್ರೆಯಿಂದ ಮತ್ತು ಇಲಾಖೆಯಿಂದ ನಿಮ್ಮ ಸಾಮಾನ್ಯ ವೈದ್ಯಕೀಯ ಆರೈಕೆಯನ್ನು ನೀವು ಮುಂದುವರಿಸುತ್ತೀರಿ. ಈ ಮಾಹಿತಿ ಹಾಳೆಯನ್ನು ಓದಲು ನಿಮಗೆ ಅವಕಾಶ ಸಿಕ್ಕ ನಂತರ ಮತ್ತು ನೀವು ಭಾಗವಹಿಸಲು ಬಯಸುತ್ತೀರಾ ಎಂದು ನಿರ್ಧರಿಸಿದ ನಂತರ, ದಯವಿಟ್ಟು ನೀವು ಏನು ನಿರ್ಧರಿಸಿದ್ದೀರಿ ಎಂಬುದನ್ನು ಸಂಶೋಧಕರಿಗೆ ತಿಳಿಸಿ. ಈ ಸಂಶೋಧನೆಯಲ್ಲಿ ಭಾಗವಹಿಸಲು ನೀವು ಒಪ್ಪಿಗೆ ಪತ್ರಕ್ಕೆ ಸಹಿ ಮಾಡಬೇಕಾಗುತ್ತದೆ, ಆದರೆ ನೀವು ಭಾಗವಹಿಸಲು ಬಯಸುತ್ತೀರೋ ಇಲ್ಲವೋ ಎಂಬುದನ್ನು ನೀವು ಸಂಶೋಧಕರಿಗೆ ತಿಳಿಸಬೇಕು. ಸಂಶೋಧನಾ ಅಧ್ಯಯನ ಅಥವಾ ಮಾಹಿತಿ ಹಾಳೆಯ ಬಗ್ಗೆ ನೀವು ಯಾವುದೇ ಪ್ರಶ್ನೆಗಳನ್ನು ಹೊಂದಿದ್ದರೆ, ದಯವಿಟ್ಟು ಸಂಶೋಧಕರನ್ನು ಕೇಳಿ.

ನಿಮ್ಮೊಂದಿಗೆ ಮನೆಗೆ ಕರೆದೊಯ್ಯಲು ಈ ಮಾಹಿತಿ ಹಾಳೆಯ ನಕಲನ್ನು ನಿಮಗೆ ನೀಡಲಾಗುವುದು. ಯಾವುದೇ ಪ್ರಶ್ನೆಗಳಿಗೆ ಅಥವಾ ಸಂದೇಹಗಳಿಗೆ ದಯವಿಟ್ಟು 09108788160 ನಲ್ಲಿ ಸಂಶೋಧಕರನ್ನು ಸಂಪರ್ಕಿಸಿ.

ನಾನು ತಿಳುವಳಿಕೆಯುಳ್ಳ ಸಮ್ಮತಿಯನ್ನು ಓದಿದ್ದೇನೆ ಮತ್ತು ಸಮೀಕ್ಷೆಯಲ್ಲಿ ಭಾಗವಹಿಸಲು ಒಪ್ಪುತ್ತೇನೆ

- ಹೌದು, ನಾನು ಸಮೀಕ್ಷೆಯಲ್ಲಿ ದಾಖಲಾಗಲು ಸಮ್ಮತಿಸುತ್ತೇನೆ (ಸ್ವಯಂಚಾಲಿತವಾಗಿ ವಿಭಾಗ 2ಗೆ ಮರುನಿರ್ದೇಶಿಸುತ್ತದೆ)
- ಇಲ್ಲ, ನಾನು ಸಮೀಕ್ಷೆಯಲ್ಲಿ ದಾಖಲಾಗಲು ಸಮ್ಮತಿಸುವುದಿಲ್ಲ (ಸಮೀಕ್ಷೆಯನ್ನು ಕೊನೆಗೊಳಿಸುತ್ತದೆ)

**ವಿಭಾಗ 2: ಜನಸಂಖ್ಯಾ ಮತ್ತು ವೈದ್ಯಕೀಯ ಡೇಟಾ**

ಸೂಚನೆಗಳು: ಪ್ರತಿ ಪ್ರಶ್ನೆಗೆ ಉತ್ತರಗಳನ್ನು ಎಚ್ಚರಿಕೆಯಿಂದ ಭರ್ತಿ ಮಾಡಿ

1. ಹೆಸರು

*ಬರೆಯುವ ಪ್ರದೇಶ*

2. ವಯಸ್ಸು (ವರ್ಷಗಳಲ್ಲಿ)

*ಬರೆಯುವ ಪ್ರದೇಶ*

3. ಲಿಂಗ

*ಪುರುಷ*

*ಹೆಣ್ಣು*

*ಇತರರು*

4. ನೀವು ತಲೆ, ಕುತ್ತಿಗೆ ಅಥವಾ ದೇಹದ ಇತರ ಭಾಗಗಳಿಗೆ ಶಸ್ತ್ರಚಿಕಿತ್ಸೆಗೆ ಒಳಗಾಗಿದ್ದೀರಾ?

*ಹೌದು*

*ಇಲ್ಲ*

5. ಹೌದು ಎಂದಾದರೆ, ವಿವರಗಳನ್ನು ನಮೂದಿಸಿ. ಇಲ್ಲದಿದ್ದರೆ, ಅದನ್ನು ಖಾಲಿ ಬಿಡಿ

*ಬರೆಯುವ ಪ್ರದೇಶ*

6. ಕಳೆದ 5 ವರ್ಷಗಳಲ್ಲಿ ನೀವು ಸ್ಟ್ರೋಕ್ ಹೊಂದಿದ್ದೀರಾ?

*ಹೌದು*

*ಇಲ್ಲ*

7. ಹೌದು ಎಂದಾದರೆ, ವಿವರಗಳನ್ನು ನಮೂದಿಸಿ. ಇಲ್ಲದಿದ್ದರೆ, ಅದನ್ನು ಖಾಲಿ ಬಿಡಿ

*ಬರೆಯುವ ಪ್ರದೇಶ*

8. ನಿಮಗೆ ಅಧಿಕ ರಕ್ತದೊತ್ತಡ, ಮಧುಮೇಹ, ಕ್ಯಾನ್ಸರ್, ದೀರ್ಘಕಾಲದ ಶ್ವಾಸಕೋಶದ ಕಾಯಿಲೆ, ಹೃದಯಾಘಾತ, ರಕ್ತ ಕಟ್ಟಿ ಹೃದಯ ಸ್ಥಂಭನ, ಆಂಜಿನಾ, ಆಸ್ತಮಾ, ಸಂಧಿವಾತ, ಪಾರ್ಶ್ವವಾಯು ಮತ್ತು ಮೂತ್ರಪಿಂಡದ ಕಾಯಿಲೆ ಇದೆ ಎಂದು ವೈದ್ಯರು ಹೇಳಿದ್ದೀರಾ?

*ಹೌದು*

*ಇಲ್ಲ*

9. ಹೌದು ಎಂದಾದರೆ, ವಿವರಗಳನ್ನು ನಮೂದಿಸಿ. ಇಲ್ಲದಿದ್ದರೆ, ಅದನ್ನು ಖಾಲಿ ಬಿಡಿ

*ಬರೆಯುವ ಪ್ರದೇಶ*

10. ನೀವು ಹೊಂದಿರುವ ಇತರ ಆರೋಗ್ಯ ಸಮಸ್ಯೆಗಳನ್ನು ಉಲ್ಲೇಖಿಸಿ (ನೀವು ಆರೋಗ್ಯವಂತರಾಗಿದ್ದರೆ ಅದನ್ನು ಖಾಲಿ ಬಿಡಿ)

*ಬರೆಯುವ ಪ್ರದೇಶ*

11. ನೀವು ಪ್ರಸ್ತುತ ಎಷ್ಟು ತೂಕ ಹೊಂದಿದ್ದೀರಿ? (ಕೆಜಿಗಳಲ್ಲಿ)

*ಬರೆಯುವ ಪ್ರದೇಶ*

12. ನೀವು ಎಷ್ಟು ಎತ್ತರ? (ಸೆಂಟಿಮೀಟರ್ ಅಥವಾ ಅಡಿಗಳಲ್ಲಿ)

*ಬರೆಯುವ ಪ್ರದೇಶ*

**ವಿಭಾಗ 2: ಈಟಿಂಗ್ ಎಸ್ಸೆಸ್ಮೆಂಟ್ ಟೂಲ್-10**

- ಸೂಕ್ತವಾದ ಪ್ರತಿಕ್ರಿಯೆಯನ್ನು ಸೂಚಿಸಿ. ಈ ಕೆಳಗಿನ ಸನ್ನಿವೇಶಗಳು ನಿಮಗೆ ಎಷ್ಟರ ಮಟ್ಟಿಗೆ ಸಮಸ್ಯೆಯಾಗಿವೆ?
- 0 ಸಂಖ್ಯೆ ಸಮಸ್ಯೆಯೇ ಇಲ್ಲ ಎಂದು ಸೂಚಿಸುತ್ತದೆ ಮತ್ತು ಸಂಖ್ಯೆಯಲ್ಲಿ ಹೆಚ್ಚಳದೊಂದಿಗೆ ನುಂಗುವ ತೊಂದರೆಯ ಮಟ್ಟವು ಹೆಚ್ಚಾಗುತ್ತದೆ, 4 ಸಂಖ್ಯೆ ಯಾವಾಗಲೂ ಸಮಸ್ಯೆ ಇರುತ್ತೆ ಎಂದು ಸೂಚಿಸುತ್ತದೆ

1. ನುಂಗುವ ಸಮಸ್ಯೆಯಿಂದಾಗಿ ನನ್ನ ತೂಕ ಕಡಿಮೆಯಾಗಿದೆ

| *0* | *1* | *2* | *3* | *4* |
| --- | --- | --- | --- | --- |
| ಸಮಸ್ಯೆಯೇ ಇಲ್ಲ |  |  |  | ತೀವ್ರ ಸಮಸ್ಯೆ |

1. ನಾನು ಹೊರ ತಿನ್ನುವ ಸಾಮರ್ಥ್ಯಕ್ಕೆ ನನ್ನ ನುಂಗುವ ಸಮಸ್ಯೆಯು ಅಡಚಣೆಯಾಗಿದೆ

| *0* | *1* | *2* | *3* | *4* |
| --- | --- | --- | --- | --- |
| ಸಮಸ್ಯೆಯೇ ಇಲ್ಲ |  |  |  | ತೀವ್ರ ಸಮಸ್ಯೆ |

1. ದ್ರವ ಪದಾರ್ಥಗಳನ್ನು ನುಂಗಲು ಹೆಚ್ಚು ಶ್ರಮ ಪಡಬೇಕಾಗತ್ತದೆ

| *0* | *1* | *2* | *3* | *4* |
| --- | --- | --- | --- | --- |
| ಸಮಸ್ಯೆಯೇ ಇಲ್ಲ |  |  |  | ತೀವ್ರ ಸಮಸ್ಯೆ |

1. ಗಟ್ಟಿ ಪದಾರ್ಥಗಳನ್ನು ನುಂಗಲು ಹೆಚ್ಚು ಶ್ರಮ ಪಡಬೇಕಾಗತ್ತದೆ

| *0* | *1* | *2* | *3* | *4* |
| --- | --- | --- | --- | --- |
| ಸಮಸ್ಯೆಯೇ ಇಲ್ಲ |  |  |  | ತೀವ್ರ ಸಮಸ್ಯೆ |

1. ಮಾತ್ರೆ / ಔಷಧಿಗಳನ್ನು ನುಂಗಲು ಹೆಚ್ಚು ಶ್ರಮ ಪಡಬೇಕಾಗತ್ತದೆ

| *0* | *1* | *2* | *3* | *4* |
| --- | --- | --- | --- | --- |
| ಸಮಸ್ಯೆಯೇ ಇಲ್ಲ |  |  |  | ತೀವ್ರ ಸಮಸ್ಯೆ |

1. ನುಂಗುವಾಗ ನನಗೆ ನೋವಾಗುತ್ತದೆ

| *0* | *1* | *2* | *3* | *4* |
| --- | --- | --- | --- | --- |
| ಸಮಸ್ಯೆಯೇ ಇಲ್ಲ |  |  |  | ತೀವ್ರ ಸಮಸ್ಯೆ |

1. ನುಂಗುವ ಸಮಸ್ಯೆಯಿಂದಾಗಿ ಅನ್ನ ಆಹಾರಗಳನ್ನು ಸೇವಿಸುವುದು ಅಷ್ಟು ಹಿತಕರವಾಗಿಲ್ಲ

| *0* | *1* | *2* | *3* | *4* |
| --- | --- | --- | --- | --- |
| ಸಮಸ್ಯೆಯೇ ಇಲ್ಲ |  |  |  | ತೀವ್ರ ಸಮಸ್ಯೆ |

1. ನುಂಗಿದಾಗ ಊಟ ಗಂಟಲಿನಲ್ಲೆ ಉಳಿಯುತ್ತದೆ

| *0* | *1* | *2* | *3* | *4* |
| --- | --- | --- | --- | --- |
| ಸಮಸ್ಯೆಯೇ ಇಲ್ಲ |  |  |  | ತೀವ್ರ ಸಮಸ್ಯೆ |

1. ನಾನು ಅನ್ನ ಆಹಾರಗಳನ್ನು ತಿಂದಾಗ ಕೆಮ್ಮುತ್ತೇನೆ

| *0* | *1* | *2* | *3* | *4* |
| --- | --- | --- | --- | --- |
| ಸಮಸ್ಯೆಯೇ ಇಲ್ಲ |  |  |  | ತೀವ್ರ ಸಮಸ್ಯೆ |

1. ನುಂಗುವುದು ಒತ್ತಡಕರವೆನಿಸುತ್ತದೆ

| *0* | *1* | *2* | *3* | *4* |
| --- | --- | --- | --- | --- |
| ಸಮಸ್ಯೆಯೇ ಇಲ್ಲ |  |  |  | ತೀವ್ರ ಸಮಸ್ಯೆ |

**ವಿಭಾಗ 2: ಡಿಸ್ಫೇಜಿಯಾ ಹ್ಯಾಂಡಿಕ್ಯಾಪ್ ಇಂಡೆಕ್ಸ್**

ನಿಮ್ಮ ಪ್ರಕಾರ ನಿಮ್ಮ ನುಂಗುವ ಸಮಸ್ಯೆಯ ಬಗ್ಗೆ ಅತ್ಯತ್ತಮವಾಗಿ ವಿವರಿಸುವ ಪದಗಳನ್ನು ಗುರುತಿಸಿ

1. ನಾನು ದ್ರವ ಪದಾರ್ಥಗಳನ್ನು ಸೇವಿಸಿದಾಗ ಕೆಮ್ಮುತ್ತೇನೆ

| *ಯಾವಾಗಲೂ ಇಲ್ಲ* | *ಕೆಲವೊಮ್ಮೆ* | *ಯಾವಾಗಲೂ* |
| --- | --- | --- |

1. ನಾನು ಘನ ಆಹಾರ ಸೇವಿಸಿದಾಗ ಕೆಮ್ಮುತ್ತೇನೆ

| *ಯಾವಾಗಲೂ ಇಲ್ಲ* | *ಕೆಲವೊಮ್ಮೆ* | *ಯಾವಾಗಲೂ* |
| --- | --- | --- |

1. ನನ್ನ ಬಾಯಿ ಒಣಗುತ್ತದೆ

| *ಯಾವಾಗಲೂ ಇಲ್ಲ* | *ಕೆಲವೊಮ್ಮೆ* | *ಯಾವಾಗಲೂ* |
| --- | --- | --- |

1. ನಾನು ಅನ್ನ ಆಹಾರಗಳನ್ನು ಸುಲಭವಾಗಿ ನುಂಗಲು ನೀರನ್ನು ಕುಡಿಯಬೇಕಾಗುತ್ತದೆ

| *ಯಾವಾಗಲೂ ಇಲ್ಲ* | *ಕೆಲವೊಮ್ಮೆ* | *ಯಾವಾಗಲೂ* |
| --- | --- | --- |

1. ನುಂಗುವ ಸಮಸ್ಯೆಯಿಂದಾಗಿ ನನ್ನ ತೂಕ ಕಡಿಮೆಯಾಗಿದೆ

| *ಯಾವಾಗಲೂ ಇಲ್ಲ* | *ಕೆಲವೊಮ್ಮೆ* | *ಯಾವಾಗಲೂ* |
| --- | --- | --- |

1. ನುಂಗುವ ಸಮಸ್ಯೆಯಿರುವುದರಿಂದ ನಾನು ಕೆಲವೊಂದು ಘನ ಆಹಾರ ಪದಾರ್ಥಗಳನ್ನು ಸೇವಿಸುವುದಿಲ್ಲ

| *ಯಾವಾಗಲೂ ಇಲ್ಲ* | *ಕೆಲವೊಮ್ಮೆ* | *ಯಾವಾಗಲೂ* |
| --- | --- | --- |

1. ನನಗೆ ತಿನ್ನಲು ಸುಲಭವಾಗುವಂತೆ ನಾನು ನುಂಗುವ ಶೈಲಿಯನ್ನು ಬದಲಾಯಿಸಿದ್ದೇನೆ

| *ಯಾವಾಗಲೂ ಇಲ್ಲ* | *ಕೆಲವೊಮ್ಮೆ* | *ಯಾವಾಗಲೂ* |
| --- | --- | --- |

1. ನುಂಗುವ ಸಮಸ್ಯೆಯಿಂದಾಗಿ ನಾನು ಸಾರ್ವಜನಿಕ ಸ್ಥಳಗಳಲ್ಲಿ ತಿನ್ನಲು ಇಷ್ಟಪಡುವುದಿಲ್ಲ

| *ಯಾವಾಗಲೂ ಇಲ್ಲ* | *ಕೆಲವೊಮ್ಮೆ* | *ಯಾವಾಗಲೂ* |
| --- | --- | --- |

1. ನನ್ನ ನುಂಗುವ ಸಮಸ್ಯೆಯಿಂದಾಗಿ ನಾನು ಊಟ ಮಾಡಲು ತೆಗೆದುಕೊಳ್ಳುವ ಸಮಯ ಮೊದಲಿಗಿಂತ ಹೆಚ್ಚಾಗಿದೆ

| *ಯಾವಾಗಲೂ ಇಲ್ಲ* | *ಕೆಲವೊಮ್ಮೆ* | *ಯಾವಾಗಲೂ* |
| --- | --- | --- |

1. ನುಂಗುವ ಸಮಸ್ಯೆಯಿಂದಾಗಿ ನಾನು ಕಡಿಮೆ ಪ್ರಮಾಣದ ಆಹಾರವನ್ನು ಆಗಾಗ ಸೇವಿಸುತ್ತೇನೆ

| *ಯಾವಾಗಲೂ ಇಲ್ಲ* | *ಕೆಲವೊಮ್ಮೆ* | *ಯಾವಾಗಲೂ* |
| --- | --- | --- |

1. ನಾನು ಅನ್ನ ಆಹಾರಗಳನ್ನು ಕೇಳಗಿಳಿಸಲು ಒಂದೆರೆಡು ಬಾರಿ ನುಂಗ ಬೇಕಾಗುತ್ತದೆ

| *ಯಾವಾಗಲೂ ಇಲ್ಲ* | *ಕೆಲವೊಮ್ಮೆ* | *ಯಾವಾಗಲೂ* |
| --- | --- | --- |

1. ನನಗೆ ಬೇಕು ಏನ್ನಿಸಿದ್ದನ್ನು ತಿನ್ನಲು ಆಗದಿರುವುದರಿಂದ ನಾನು ಖಿನ್ನತೆಗೆ ಒಳಗಾಗುತ್ತೇನೆ

| *ಯಾವಾಗಲೂ ಇಲ್ಲ* | *ಕೆಲವೊಮ್ಮೆ* | *ಯಾವಾಗಲೂ* |
| --- | --- | --- |

1. ಇತ್ತೀಚೆಗೆ ನಾನು ತಿನ್ನುವುದನ್ನು ಮೊದಲಿನಷ್ಟು ಇಷ್ಟಪಡುತ್ತಿಲ್ಲ

| *ಯಾವಾಗಲೂ ಇಲ್ಲ* | *ಕೆಲವೊಮ್ಮೆ* | *ಯಾವಾಗಲೂ* |
| --- | --- | --- |

1. ನುಂಗುವ ಸಮಸ್ಯೆಯಿಂದಾಗಿ ನಾನು ಜನರೊಂದಿಗೆ ಹೆಚ್ಚಾಗಿ ಬೆರೆಯುತ್ತಿಲ್ಲ

| *ಯಾವಾಗಲೂ ಇಲ್ಲ* | *ಕೆಲವೊಮ್ಮೆ* | *ಯಾವಾಗಲೂ* |
| --- | --- | --- |

1. ನುಂಗುವ ಸಮಸ್ಯೆಯಿಂದಾಗಿ ನಾನು ತಿನ್ನುವುದರಿಂದ ದೂರ ಉಳಿಯುತ್ತೇನೆ

| *ಯಾವಾಗಲೂ ಇಲ್ಲ* | *ಕೆಲವೊಮ್ಮೆ* | *ಯಾವಾಗಲೂ* |
| --- | --- | --- |

1. ನುಂಗುವ ಸಮಸ್ಯೆಯಿಂದಾಗಿ ನಾನು ಕಡಿಮೆ ತಿನ್ನುತ್ತೇನೆ

| *ಯಾವಾಗಲೂ ಇಲ್ಲ* | *ಕೆಲವೊಮ್ಮೆ* | *ಯಾವಾಗಲೂ* |
| --- | --- | --- |

1. ನುಂಗುವ ಸಮಸ್ಯೆಯಿಂದಾಗಿ ನಾನು ಒತ್ತಡಕ್ಕೆ ಒಳಗಾಗುತ್ತೇನೆ

| *ಯಾವಾಗಲೂ ಇಲ್ಲ* | *ಕೆಲವೊಮ್ಮೆ* | *ಯಾವಾಗಲೂ* |
| --- | --- | --- |

1. ನುಂಗುವ ಸಮಸ್ಯೆಯಿಂದಾಗಿ ನಾನು ಅಸಮರ್ಥನಾಗಿದ್ದೇನೆ ಎಂದು ಅನಿಸುತ್ತದೆ

| *ಯಾವಾಗಲೂ ಇಲ್ಲ* | *ಕೆಲವೊಮ್ಮೆ* | *ಯಾವಾಗಲೂ* |
| --- | --- | --- |

1. ನನ್ನ ನುಂಗುವ ಸಮಸ್ಯೆಯಿಂದಾಗಿ ನನ್ನ ಮೇಲೆ ನನಗೆ ಕೋಪ ಬರುತ್ತದೆ

| *ಯಾವಾಗಲೂ ಇಲ್ಲ* | *ಕೆಲವೊಮ್ಮೆ* | *ಯಾವಾಗಲೂ* |
| --- | --- | --- |

1. ನಾನು ಔಷದಿಗಳನ್ನು ಸೇವಿಸುವಾಗ ನೆತ್ತಿಗೇರುತ್ತದೆ

| *ಯಾವಾಗಲೂ ಇಲ್ಲ* | *ಕೆಲವೊಮ್ಮೆ* | *ಯಾವಾಗಲೂ* |
| --- | --- | --- |

1. ನನ್ನ ನುಂಗುವ ಸಮಸ್ಯೆಯಿಂದಾಗಿ ನನಗೆ ನೆತ್ತಿಗೆತ್ತಿ ಉಸಿರುಕಟ್ಟುತ್ತದೆ ಎಂದು ಭಯವಾಗುತ್ತದೆ

| *ಯಾವಾಗಲೂ ಇಲ್ಲ* | *ಕೆಲವೊಮ್ಮೆ* | *ಯಾವಾಗಲೂ* |
| --- | --- | --- |

1. ನನ್ನ ನುಂಗುವ ಸಮಸ್ಯೆಯಿಂದಾಗಿ ನಾನು ಬೇರೆ ರೀತಿಯಲ್ಲಿ (ಕೊಳವೆಯ ಸಹಾಯದಿಂದ) ತಿನ್ನಬೇಕಾಗುತ್ತದೆ

| *ಯಾವಾಗಲೂ ಇಲ್ಲ* | *ಕೆಲವೊಮ್ಮೆ* | *ಯಾವಾಗಲೂ* |
| --- | --- | --- |

1. ನುಂಗುವ ಸಮಸ್ಯೆಯಿಂದಾಗಿ ನನ್ನ ಆಹಾರ ಶೈಲಿಯು ಬದಲಾಗಿದೆ

| *ಯಾವಾಗಲೂ ಇಲ್ಲ* | *ಕೆಲವೊಮ್ಮೆ* | *ಯಾವಾಗಲೂ* |
| --- | --- | --- |

1. ನುಂಗುವಾಗ ನನಗೆ ಉಸಿರುಕಟ್ಟಿದಂತೆ ಅನಿಸುತ್ತದೆ

| *ಯಾವಾಗಲೂ ಇಲ್ಲ* | *ಕೆಲವೊಮ್ಮೆ* | *ಯಾವಾಗಲೂ* |
| --- | --- | --- |

1. ನಾನು ನುಂಗಿದ ನಂತರ ಕೆಮ್ಮಿ ಆಹಾರವನ್ನು ಹೊರಗೆ ಹಾಕುತ್ತೇನೆ

| *ಯಾವಾಗಲೂ ಇಲ್ಲ* | *ಕೆಲವೊಮ್ಮೆ* | *ಯಾವಾಗಲೂ* |
| --- | --- | --- |

1. ನಿಮ್ಮ ಪ್ರಕಾರ ನಿಮ್ಮ ನುಂಗುವ ಸಮಸ್ಯೆಯ ಪ್ರಮಾಣವನ್ನು ಹೋಲುವ ಸಂಖ್ಯೆಯನ್ನು ಗುರುತಿಸಿ (1= ತೊಂದರೆಯೇ ಇಲ್ಲ, 4=ಸ್ವಲ್ಪ ಹೆಚ್ಚು ಸಮಸ್ಯೆ, 7=ತೀವ್ರ ಸಮಸ್ಯೆ)

| 1 | 2 | 3 | 4 | 5 | 6 | 7 |
| --- | --- | --- | --- | --- | --- | --- |
| *ತೊಂದರೆಯೇ ಇಲ್ಲ* | | *ಸ್ವಲ್ಪ ಹೆಚ್ಚು ಸಮಸ್ಯೆ* | | | *ತೀವ್ರ ಸಮಸ್ಯೆ* | |
